# Development of multiplex PCR to detect slow rust resistance genes *Lr34* and *Lr46* in wheat

**DOI:** 10.1007/s13353-019-00520-z

**Published:** 2019-09-10

**Authors:** Roksana Skowrońska, Michał Kwiatek, Agnieszka Tomkowiak, Jerzy Nawracała

**Affiliations:** Department of Genetics and Plant Breeding, Faculty of Agronomy and Bioengineering, Poznań University of Life Sciences, 11 Dojazd Str, 60-632 Poznań, Poland

**Keywords:** Leaf rust, *Lr34*, *Lr46*, Multiplex PCR, Wheat

## Abstract

Leaf rust caused by *Puccinia triticina* belongs to one of the most dangerous fungal diseases of wheat (*Triticum aestivum* L.) and is the cause of large yield losses every year. Here we report a multiplex polymerase chain reaction (PCR) assay, which was developed for detection of two important wheat slow rust resistance genes *Lr34* and *Lr46*, using two molecular markers: *csLV34* and *Xwmc44*, respectively. The presence of genes was analyzed in one winter wheat variety TX89D6435 and five spring wheat varieties: Pavon F76, Parula ‘S’, Rayon 89, Kern, Mochis 88. Both *Lr34* and *Lr46* genes were identified in variety TX89D6435, gene *Lr34* was also identified in Parula ‘S’ and Kern varieties, and gene *L46* occurs in Pavon F76 and Mochis 88 variety. None of the resistance genes tested was detected in the Rayon 89 variety. The use of the multiplex PCR method allowed to shorten the analysis time, reduce costs of analyses, and reduce the workload.

## Introduction

Leaf rust caused by the pathogen *Puccinia triticina* Erikss. & Henn. is one of the most destructive diseases of wheat (Kolmer et al. 2005). There are many fungicides that help control these fungal disease, but their use is expensive and can have a negative impact on the environment. The most efficient, economical, and environmentally sound method to mitigate the losses caused by pathogens is breeding for genetic resistance (Muthe et al. 2016). To date, more than 70 leaf rust resistance (*Lr*) genes have been cataloged (McIntosh et al. 2013), but many of these genes are race-specific and they have lose their effectiveness when new races of the pathogen are appearing (McCallum et al. 2007). Currently, breeding programs focus on producing cultivars with adult plant resistance (APR), also known as slow rusting or race non-specific genes. Slow rusting genes are a group of leaf rust resistance genes that give durable resistance only in adult plant (Bošković et al. 2008). APR is characterized by less and slower pathogen growth and reproduction despite a high infection type (Tiwari et al. 2009). An important advantage of genes is their pleiotropic effect on many pathogens, for example *Blumeria graminis* causing powdery mildew (Lillemo et al. 2008). Among all, the leaf rust resistance genes in wheat only four genes are known as slow rusting: *Lr34* (Singh 1992), *Lr46* (Singh et al. 1998), *Lr67* (Dyck and Samborski 1979), and *Lr68* (Herrera-Foessel et al. 2012).

The leaf rust resistance gene *Lr34* (earlier *LrT2*) is the best known and most effective of slow rusting genes. The gene *Lr34* was first described in the wheat line PI58548 and located on short arm of wheat chromosome 7D (Dyck 1987). Lagudah et al. (2006) developed PCR-based marker *csLV34* that has been used extensively to identify the presence of *Lr34* gene. The disadvantage is that the marker is not diagnostic in some genetic backgrounds, like Canadian wheat germplasm (Lagudah et al. 2009). Leaf tip necrosis (LTN) is one of the morphological markers associated with leaf rust resistance gene *Lr34* and is also expressed in the absence of the pathogen (Lagudah et al. 2006). The *Lr46* gene was first described in wheat cultivar Pavon F76 and was localized on chromosome 1BL (Singh et al. 1998). *Lr46* shows a resistance phenotype in adult plants similar to *Lr34*, but the effects of *Lr46* are not as pronounced as *Lr34* (Martinez et al. 2001). Lillemo et al. (2008) have shown that *Lr46* has an additive effect on leaf rust resistance of *Lr34*. The gene *Lr46* was mapped distal to *Xwmc44* and proximal to *Xgwm259* (Suenaga et al. 2003).

## Material and methods

The aim of this study was to develop a multiplex PCR method for simultaneous identification of two most effective slow rust resistance genes: *Lr34* and *Lr46*. Plant material consisted of six wheat cultivars *Triticum aestivum* L. derived from the National Small Grains Collection, the Agriculture Research Station in Aberdeen, USA: TX89D6435, Pavon F76, Parula ‘S’, Rayon 89, Kern and Mochis 88 (Table 1).

**Table 1:** Presence of genes *Lr34* and *L46* in tested wheat varieties

No.	Cultivar	Origin	Plant ID	Presence of Lr34	Presence of Lr46
1.	TX89D6435	US, Texas	PI 584759	+	+
2.	Pavon F76	Mexico	PI 520003	–	+
3.	Parula ‘S’	Mexico	PI 520340	+	–
4.	Rayon 89	Mexico	PI 591784	–	–
5.	Kern	US	PI 672001	+	–
6.	Mochis 88	Mexico	PI 591791	–	+

The DNA was extracted from leaf tissue using the GeneMATRIX Plant & Fungi DNA Purification Kit (EURx Ltd., Poland). DNA quality and concentration was checked using the DeNovix spectrophotometer. In order to identify the *Lr34* and *Lr46* genes, two molecular markers were used: *csLv34* and *Xwmc44*. The STS marker *csLV34* maps 0.4 cM from *Lr34* and the sequence of primers (Merck) is as follows: csLV34F 5′- GTT GGT TAA GAC TGG TGA TGG -3′; csLV34R 5′- TGC TTG CTA TTG CTG AAT AGT -3′ (Lagudah et al. 2006). According to the literature, the size of the amplified product is a 150 bp (base pairs) band, indicative of the presence of the gene and a 229 bp band in susceptible genotypes. Locus of SSR marker *Xwmc44* is located 0.4 cm from QTL for *Lr46*. A product of the microsatellite marker is 242 bp band for the presence of *Lr46* gene, and the sequence of marker primers is as follows: WMC44F 5′- GGT CTT CTG GGC TTT GAT CCT G -3′, WMC44R 5′- GTT GCT AGG GAC CCG TAG TGG -3′ (Suenaga et al. 2003). The 25 μL mix composition of multiplex PCR volume consisted of the following: 12.5 μL 2× PCR TaqNovaHs PCR Master Mix (Blirt), which included 2× concentrated PCR reaction buffer, 4 mM MgCl_2_; 1.6 mM dNTPs mix (0.4 mM of each dNTP); 0.8 μL *csLv34* forward primer; 0.8 μL *csLv34* reverse primer; 1.2 μL *Xwmc44* forward primer; 1.2 μL *Xwmc44* reverse primer (the concentration for each primer was 100 μM); 2 μL DNA template (50 ng/μL) and 6.5 μL PCR grade water. PCR profile was modified with reference to standard protocol. The following annealing temperatures were tested: 55 °C, optimal for the *csLv34* primer (Lagudah et al. 2006), 61 °C recommended for the *Xwmc44* marker (Suenaga et al. 2003), and several intermediate variants. The final PCR reaction consisted of initial denaturation at 94 °C for 5 min, followed by 40 cycles (denaturation, 94 °C for 45 s; primer annealing, 60 °C for 30 s; elongation, 72 °C for 1 min), followed by the final extension for 7 min at 72 °C and final step at 4 °C. The reaction was carried out using the Labcycler thermal cyclers (SensoQuest GmbH). The products of amplification were prepared by adding 0.5 Midori Green Direct (NIPPON Genetics EUROPE) to each tube and were separated using 2% agarose (SIGMA) gel in 1× TBE buffer (BioShop) at 100 V for two and a half hours. A Molecular Imager Gel Doc™ XR UV system was used with the Biorad Bio Image™ Software to visualize the PCR products.

## Results and discussion

**Fig. 1:**
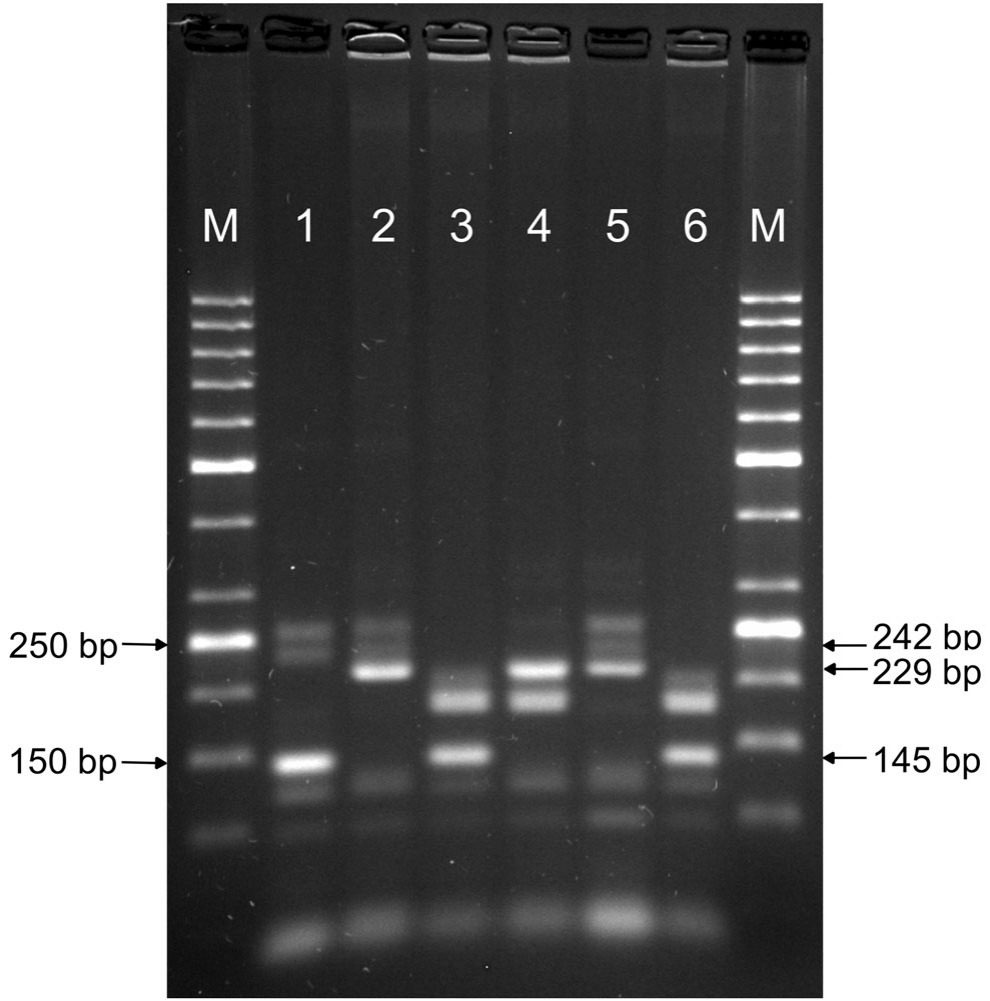
Electrophoresis showing the presence of markers: *csLv34* (for the *Lr34*) and *Xwmc44* (for the *Lr46*) in the wheat varieties. M, GeneRuler 50 bp DNA ladder (NIPPON Genetics EUROPE GmbH); 1, TX89D6435; 2, Pavon F76; 3, Parula ‘S’; 4, Rayon 89; 5, Kern; 6, Mochis 88

Molecular markers can be successfully used in the identification of leaf rust resistance genes in wheat resistance breeding programs (Vida et al. 2009). The results showed that the amplification of *csLv34* marker was observed in TX89D6435, Parula ‘S’, and Kern varieties, but the size of the resulting *Lr34* gene linked product was approximately 145 bp, which is smaller than reported by Lagudah et al. (2006). Differences in the size of products may result from the size of the DNA ladder used. Lagudah et al. (2006) used a 100 bp ladder molecular size markers. In our experiment we have used more precise, 50 bp DNA ladder, which showed that the *csLv34* marker product is smaller than 150 bp. Considering other varieties (Pavon F76, Rayon 89, and Kern’) the PCR reaction with *csLv34* marker showed a 229 bp product, indicating the lack of the *Lr34* gene. The analyses with the *Xwmc44* marker linked to the *Lr46* gene resulted in the identification of a 242 bp specific product in TX89D6435, Pavon F76, and Mochis 88 varieties. The accumulation of both *Lr34* and *Lr46* resistance genes was demonstrated by the multiplex PCR in TX89D6435 variety, so this variety can be a good source of non-race specific resistance to leaf rust (Table 1, Fig. 1).

The literature gives many examples of successful use of the multiplex PCR method to identify race-specific resistance genes. Leśniowska-Nowak et al. (2013) developed a multiplex PCR method to identify two resistance genes for leaf rust *Lr9* and *Lr19*. Other major resistance genes for *P. triticin*a *Lr29* and *Lr37* were identified with one PCR reaction by Sumikova and Hanzalova (2010). Gogół et al. (2015) made a successful attempt to use the multiplex PCR method to simultaneously identify genes of resistance to two different diseases: *Lr21* (leaf rust) and *Pm4b* (powdery mildew). Tomkowiak et al. (2019) identified the *Pm2*, *Pm3a*, *Pm4b*, and *Pm6* genes and developed multiplex PCR reaction conditions for simultaneous identification of *Pm2* and *Pm4b* genes.

Slow rusting genes *Lr34* and *Lr46* are very important for breeding because they provide durable resistance over a long period of time in different environments, and they are effective against many pathogens (Imbaby et al. 2014). The development of the multiplex PCR method allows to significantly shorten the time of analysis of these two important genes. The study demonstrated that the developed multiplex PCR conditions are effective diagnostic tool for the simultaneous identification of *Lr34* and *L46* genes using the *csLv34* and *Xwmc44* markers, respectively. The developed multiplex PCR conditions can be used in breeding programs for marker-assisted selection.
